# Linking Work Values Profiles to Basic Psychological Need Satisfaction and Frustration

**DOI:** 10.1177/00332941211040439

**Published:** 2021-08-14

**Authors:** Mathieu Busque-Carrier, Catherine F. Ratelle, Yann Le Corff

**Affiliations:** Département d’orientation professionnelle, 7321 Faculté d’éducation, Université de Sherbrooke, Canada; Département des fondements et pratiques en éducation, 4440 Faculté des sciences de l’éducation, Université Laval, Canada; Département d’orientation professionnelle, 7321 Faculté d’éducation, Université de Sherbrooke, Canada

**Keywords:** Work values, need satisfaction at work, need frustration at work, latent profiles, person-centered approach

## Abstract

The association between work values and key motivational variables has been repeatedly supported in previous studies. However, little attention has been devoted to understanding intraindividual patterns of work values and how combinations of work values relate to other motivational variables. This study aimed to identify profiles of work values based on a four-factor model (i.e., intrinsic, extrinsic, social, and status). It also investigated how profile membership relates to basic psychological need satisfaction and frustration at work using a self-determination perspective. A sample of French Canadian adults (N = 476) participated in this study by filling out an online questionnaire. Latent profile analyses revealed five distinct work values profiles. Results showed that participants in more positive profiles (i.e., high level of intrinsic, social, and status work values) generally reported higher level of need satisfaction and lower level of need frustration at work than participants belonging to more negative profiles (i.e., low level of intrinsic, social, and status work values). These results support the importance of considering work values in organizational and career development interventions, and to do so using a person-centered approach, to better understand need satisfaction and frustration at work.

## Introduction

Work values are defined as motivational beliefs specific to the career context that serve as criteria or orientations for assessing jobs and work environments (Ros et al., 1999; Super, 1980). Work values are associated to several vocational covariates like work satisfaction (Knoop, 1994; Moniarou-Papaconstantinou & Triantafyllou, 2015), career choice (Balsamo et al., 2013; Judge & Bretz, 1992), work decision-making (Knoop, 1991), basic psychological needs at work (Vansteenkiste et al., 2007), boredom proneness (Vodanovich et al., 1997), career adaptability (Ye, 2015), work engagement (Sortheix et al., 2013), and life satisfaction (Chow et al., 2017; Vansteenkiste et al., 2007). While several studies examined how each factor of work values are associated with these outcomes, few studies investigated the contribution of all work values simultaneously. That is, when examining the association between a specific work values factor and an outcome, individuals’ endorsement of other factors are typically not taken into account. However, values do not exist in a vacuum and considering the whole work values system when assessing their contribution to motivational covariates should substantially improve our understanding of the contributions of work values. One approach that allows considering intraindividual combinations of a set of variables such as work values is the identification of profiles (i.e., subpopulations characterized by a similar configuration on a set of variables such as work values) and how these profiles are related to important outcomes (Marsh et al., 2009). Identifying work values profiles can lead to the development of more adapted and specific interventions with subpopulations of individuals (Howard et al., 2016; Morin & Marsh, 2015).

To our knowledge, very few studies have tried to identify work values profiles, and none have done so using a four-factor model (i.e., intrinsic, extrinsic, social, and status). Estimating work values profiles using a four-factor model rather than two- (Vansteenkiste et al., 2007) or three-factor models (Macnab et al., 2005; Manhardt, 1972; Super, 1970) should be favored in light of previous findings showing that each of the four factors had a unique association with key constructs (e.g., personality traits, vocational interests, educational aspirations; Duffy & Sedlacek, 2007; Hirschi, 2008; Wöhrmann et al., 2016 ), had a distinctive developmental pattern (Jin & Rounds, 2012), and offered a superior fit to the data when compared to other models (Busque-Carrier et al., 2021a). Hence, this study sought to identify profiles of work values using a four-factor model.

## Model of work values

The four-factor model of work values (Busque-Carrier et al., 2021a) includes 15 specific work values that are grouped under four factors: intrinsic, extrinsic, social, and status work values. The *intrinsic* factor groups work values for which the source of satisfaction is inherent to the tasks accomplished at work (Ros et al., 1999). Five work values are included in this factor: autonomy, creativity, development, intellectual stimulation, and variety. The *extrinsic* factor is composed of work values for which the source of satisfaction comes from consequences or rewards obtained by working (Nevill and Kruse, 1996). Four extrinsic work values are included in this factor: income, security, work environment, and work-life balance. The *social* factor includes two work values (i.e., altruism and supervisors) for which the source of satisfaction comes from significant and meaningful work relationships (Macnab et al., 2005). The *status* factor encompasses work values that promote personal success and a desire to manage others. Status work values identified in the model are advancement, authority, recognition, and travel. Definitions of each work value are presented in Table S1 of the online supplements. Readers are referred to Busque-Carrier et al. (2021a) for additional discussions about the FFM-WV and how this model is different from other work values models in the literature.

**Figure 1.**
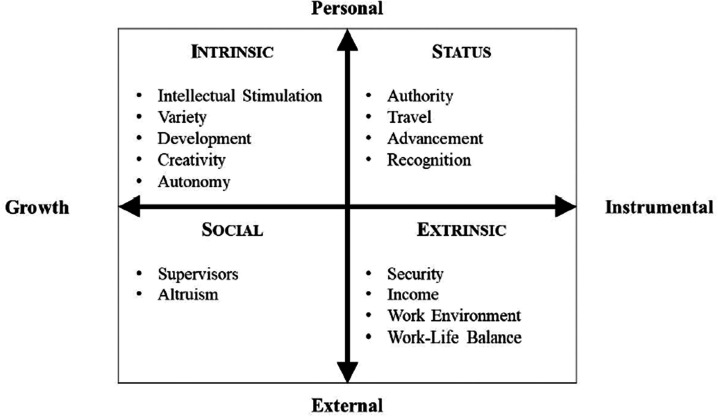
The four-factor model of work values. *Note*. Each work value factor refers to a quadrant.

In this model, factors of work values can be organized according to two axes (see Figure 1). The first axis (i.e., horizontal axis in Figure 1) is used to differentiate work values based on their content, where they can be either growth-oriented (i.e., intrinsic, social) or instrumental (i.e., status, extrinsic). The second axis (i.e., vertical axis in Figure 1) refers to the orientation (or interest) the value endorsement serves, where values can be either personally (i.e., intrinsic, status) or externally (i.e., social, extrinsic) oriented. To better understand the contribution of work values factors to important work variables such as the state of workers psychological needs, the FFM-WV relies on a self-determination perspective.

## A self-determination perspective on values

Self-determination theory (SDT; Ryan and Deci, 2017) is an organismic-dialectical theory that conceptualizes optimal psychological functioning and development as resulting from the combined interplay between individuals’ innate characteristics and their social environment. SDT postulates that all individuals have three innate psychological needs – autonomy, competence, and relatedness – whose satisfaction underlies psychological thriving. When these needs are satisfied, individuals experience a sense of self-endorsement and ownership of their actions and behaviors (autonomy satisfaction; de Charms, 1968; Deci & Ryan, 2000), feel capable and effective in their actions (competence satisfaction; Deci & Ryan, 2000; White, 1959), and experience meaningful connections and closeness with important individuals (relatedness satisfaction; Baumeister & Leary, 1995; Deci & Ryan, 2000). In contrast, when these needs are frustrated, individuals feel controlled by external or self-imposed pressures (autonomy frustration), uncapable or ineffective in their actions (competence frustration), and excluded by valued others (relatedness frustration; Rouse et al., 2020). Whereas psychological need satisfaction (PNS) is related to greater well-being and to positive outcomes (e.g., life satisfaction, vitality, positive affect), psychological need frustration (PNF) is associated with negative outcomes for human functioning, like depressive symptoms, stress, or anxiety (for a review, see Rouse et al., 2020). PNS and PNF have been contextualized to different life domains (e.g., work, school, family, and sports) and robust support exist for their contribution to, respectively, positive and negative outcomes in these contexts (Deci and Ryan, 2008; Ryan & Deci, 2017).

According to SDT, all values are not created equal nor contribute similarly to psychological growth and wellness (Kasser, 2002; Ryan et al., 1996). Specifically, types of values can be distinguished based on their association with PNS and PNF (Vansteenkiste et al., 2007; Vansteenkiste & Ryan, 2013). This distinctfion was also made for work values in previous studies, which showed that intrinsic and social work values were positively related to PNS at work and negatively to PNF at work, whereas extrinsic and status work values were positively associated to PNF at work and negatively to PNS at work  (Busque-Carrier et al., 2021b; Schreurs et al., 2014; Vansteenkiste et al., 2007; Zhang et al., 2019). Some studies examined the association of work values with PNS by creating a score that represent the relative contribution of one value factor, to partially control the contribution of other factors. Hence, individuals who find the work domain important are expected to value every factor of work values highly because they are all connected to work (Schreurs et al., 2014). For example, Vansteenkiste and his colleagues (2007) assessed the extrinsic, relative to intrinsic, work value construct by using an extrinsic work value score as a predictor, after controlling for overall work importance (i.e., the sum of every work values scores). Results showed that endorsing extrinsic work values more strongly than intrinsic work values was negatively related to PNS at work. To our knowledge, the association between the relative contribution of work values and PNF at work has yet to be studied. However, examining the relative contribution of work values with the four-factor model of work values is far from optimal, because it neglects the multidimensionality of work values – i.e., the contribution of every work values factor simultaneously. It does not allow to detect complex interactions among the four factors. Rather than accounting for the combined contribution of every work value, a relative score represents the specific contribution of a factor after excluding the contribution of every factor together. Recent statistical developments make it possible to consider how all values combine within the self, which requires using a person-centered approach.

## A person-centered approach to work values

A person-centered approach offers an alternative way to study relationships among variables of interest by identifying subgroups within a sample who differ in quantity (low to high levels) and quality (e.g., intrinsic, extrinsic, social, and status) of work values. In other words, person-centered analyses provide information on how a group of variables coexist within individuals and how subgroups of individuals display unique patterns of combination (Meyer et al., 2013). Applied to work values, subpopulations are identified based on typical configurations of work values factors. Profile membership can be associated with variables of interest such as PNS and PNF.

To our knowledge, only two studies identified work values profiles. A first study investigated work values profiles in a sample of American lawyers using a two-factor model of work values (intrinsic and extrinsic work values; Koh, 2016). Five profiles were obtained, with four profiles distinguishable quantitatively (i.e., profiles with low to high levels for both work values) and one profile characterized by contrasting levels of work values (i.e., high intrinsic–low extrinsic). The second study (Guo et al., 2018) identified work values profiles in a sample of Finnish teenagers using a five-factor model of work values that included two social work values (i.e., working with others and contribution to society), two extrinsic work values (i.e., income and family), and one status work values (i.e., career advancement). This study identified four work values profiles characterized by: (1) strong endorsement of extrinsic-income and weak endorsement of social work values; (2) strong endorsement of status work value and low endorsement of extrinsic-family work value; (3) moderate level of extrinsic-family work values and low level of status, and (4) high levels of social work values and low level of extrinsic-income. This study also examined the extent to which these profiles differed based on gender. Results showed that women were over-represented in profiles characterized by a stronger endorsement of social than of extrinsic and status work values (i.e., profiles 3 and 4), whereas men were over-represented in profiles characterized by higher levels of extrinsic-income valuing versus social work values (i.e., profile 1). To our knowledge, profile differentiation based on other sociodemographic variables like age has not yet been examined.

In sum, the reviewed literature yields three conclusions. First, examining factors of work values can inform on the state of workers’ psychological needs. Specifically, when individuals strongly endorse growth-oriented work values (intrinsic, social), they report their needs for autonomy, competence, and relatedness as being more satisfied and less frustrated at work. In contrast, when individuals strongly endorse instrumentally-oriented work values (extrinsic, status), they report their needs for autonomy, competence, and relatedness as being more frustrated and less satisfied at work. Second, previous studies (Guo et al., 2018; Koh, 2016) showed that it is possible to identify unique profiles of work values to examine how subgroups of individuals display unique patterns of values combination (Meyer et al., 2013). Third, work values profiles have yet to be identified using the FFM-WV. Therefore, research is needed to identify profiles of work values with the FFM-WV and examine whether these profiles can be distinguished on measures of PNS and PNF at work, which are respectively important contributors to positive and negative outcomes for human functioning at work.

## The present study

The goal of this study was to identify work values profiles and examine their relations with PNS and PNF at work. This research expands the literature on work values by (1) including all four factors of work values in a same model; (2) using a sample of workers from a variety of job domains; and (3) relating profile membership with two motivational processes (i.e., PNS and PNF at work). Two specific objectives were pursued. The first objective was to identify work values profiles using latent profile analysis, based on the FFM-WV. We expected a small number of work values profiles (i.e., four or five), based on previous studies that found four or five profile solutions to be optimal even though they relied on different conceptualizations of work values (Guo et al., 2018; Koh, 2016). However, given that this study is only the third to apply a person-centered approach to work values and the first to do so with a four-factor model, this hypothesis remain preliminary and not specifically tested. The second goal of this study was to associate membership to work values profiles with measures of PNS and PNF at work. Based on previous studies and SDT propositions, we expected that participants belonging to profiles characterized by high levels of intrinsic and social work values and low levels of extrinsic and status will report higher levels of PNS at work and lower PNF at work. We also expected participants in profiles characterized by low levels of intrinsic and social work values and high levels of extrinsic and status to report higher levels of PNF at work and lower PNS at work. Considering that previous studies showed that some work values factors were related to age (e.g., Jin & Rounds, 2012) and gender, (e.g., Sortheix et al., 2013) profile membership was also examined as a function of these sociodemographic variables, although no specific hypothesis was formulated.

## Method

### Participants and procedure

The sample included 476 workers (63% female; 26% men; 11% unspecified) aged between 20 and 68 years (M = 43; *SD* = 9.5) from a governmental organization. Most participants held a full-time job (87%), were born in the province of Quebec (83%), and spoke French at home (99%). Participants mainly worked in government and public administration (58%) or in business management and administration (13%) work domains. Median annual family income was above $100,000 CAN, which is higher than the median household income in Quebec (Statistics Canada, 2016). All participants earned a high school diploma and more than half of them (56%) earned a university degree. Participants cumulated an average of 16.1 years of schooling (*SD* = 3.9). During the fall of 2019, they received an email from their human resources department inviting them to participate in this study. Participants filled a consent form and completed an online questionnaire assessing work values, basic psychological needs at work, and sociodemographic information. Both consent form and questionnaire were hosted on a secure, university-based server. Data were collected and treated with approval from the Ethics Committee of the first author’s home university.

### Measures

#### Work values

The Integrated Work Values Scale (IWVS; Busque-Carrier et al., 2021a) is a 70-item French scale assessing the 15 specific work values constituting the FFM-WV. Participants indicated the importance they attach to different criteria or orientations related to jobs or work environments using a 5-point Likert-type scale varying from 1 (*not important at all*) to 5 (*very important*). Subscales of the IWVS are grouped into four work values factors: intrinsic (24 items; e.g. “At work, it is important for me to be able to improve my abilities”), extrinsic (17 items; e.g. “At work, it is important for me to have a good salary”), social (10 items; e.g. “At work, it is important for me to be of service to others”), and status (19 items; e.g. “At work, it is important for me to be recognized for the work tasks that I accomplished”). A previous study supported the psychometric properties of the IWVS (see Busque-Carrier et al., 2021a). In the present study, omega coefficients were all satisfactory (ranging from .84 to .88; see Table 1).

**Table 1.** Means, standard deviations, omega coefficients and correlations among scores (N = 476).

	M	SD	1	2	3	4	5	6	7	8
1. Intrinsic Work Values	0.00	0.52	*.86*							
2. Social Work Values	0.00	0.46	.70^**^	*.84*						
3. Extrinsic Work Values	0.00	0.58	.07	.60^**^	*.84*					
4. Status Work Values	0.00	0.37	.83^**^	.63^**^	.42^**^	*.88*				
5. Need Satisfaction	0.00	0.92	.20	.15^**^	−.03	.11^*^	*.81*			
6. Need Frustration	0.00	0.94	−.07	−.11^*^	.03	.02	−.77^**^	*.87*		
7. Gender^a^	1.30	0.47	−.04	−.14^**^	−.09	.00	−.06	.13^**^		
8. Age	43.13	9.45	−.04	−.05	−.07	−.12^*^	−.04	.03	.12^*^	
9. Years of schooling	16.11	3.87	.12^*^	−.03	−.15^**^	.05	−.01	.07	.08	.09

*Note*. Work values and psychological needs scores are factor scores obtained from preliminary measurement models. Composite reliability estimates (ω) are reported in *italics* on the diagonal.

^a^Women = 1, Men = 2.

*p < .05, **p < .01.

#### Basic psychological needs at work

The French version (Chevrier & Lannegrand-Willems, 2018) of the Basic Psychological Need Satisfaction and Frustration Scale (BPNSFS; Chen et al., 2015) was used to assess satisfaction and frustration of basic psychological needs at work. The label “At work” was added to contextualize the items to the work context. Participants indicated the extent to which they agreed with 24 items using a 5-point Likert-type scale ranging from 1 (*not true at all*) to 5 (*completely true*). A total of 12 items were grouped to yield a score of PNS at work, covering autonomy (4 items; e.g. “At work, I feel a sense of choice and freedom in the things I undertake”), relatedness (4 items; e.g. “At work, I feel that the people I care about also care about me.”), and competence satisfaction (4 items; e.g. “At work, I feel confident that I can do things well.”). Similarly, 12 items were grouped to yield a score of PNF at work, covering autonomy (4 items; e.g. “I feel pressured to do too many things”), relatedness (4 items; e.g. “At work, I feel the relationships I have are just superficial”), and competence (4 items; e.g. “At work, I feel like a failure because of the mistakes I make”) frustration. The BPNSFS was found to be reliable and valid in past research (Chen et al., 2015; Tóth-Király et al., 2018) and in this study, where omega coefficients were .81 for PNS at work subscale and .87 for PNF at work subscale.

#### Sociodemographic information

Participant answered questions regarding their age, gender, language spoken at home, birthplace, family income, job domain, highest level of education, and years of schooling.

### Statistical analyses

#### Model estimation

Models were estimated using Mplus 8.4 (Muthén & Muthén, 2019) under robust Maximum Likelihood (MLR) estimator, which provides fit indices and standard errors that are robust to the non-normality of the data and to the Likert nature of the items (Hair et al., 2010). Model fit was assessed with the Comparative Fit Index (CFI), the Tucker-Lewis Index (TLI), the Root Mean Square Error of Approximation (RMSEA), and the Standardized Root Mean Square Residual (SRMR). According to typical interpretation guidelines (e.g., Marsh et al., 2005), values greater than .90 for the CFI and TLI and smaller than .08 for the RMSEA and SRMR are considered to indicate adequate fit to the data whereas values greater than .95 for the CFI and TLI and smaller than .06 for the RMSEA and SRMR indicate an excellent model fit. Missing data for each item of work values (ranging from 0% to 2%) and basic psychological needs at work (ranging from 9% to 11%) were accommodated via the full information maximum likelihood estimation (Enders, 2010), which is a better alternative than mean substitution or case deletion.

#### Measurement models

The factor structure of each variable was assessed to make sure that the expected theoretical model has good fit to the data. Factor scores were then extracted from these measurement models, which are scores that preserve the underlying nature of the latent constructs. They are superior to observed means, because they partially control measurement errors by giving less weight to items with lower factor loadings (Skrondal & Laake, 2001). Furthermore, factor scores are standardized (i.e., mean = 0, standard deviation = 1), which makes them directly comparable and easy to interpret.

Factor scores for work values factors were generated based on an ESEM-within-CFA model (EwC; Marsh et al., 2013; Morin & Asparouhov, 2018). EwC are used to estimate a hierarchical model where the first-order structure is assessed using an exploratory structural equation modeling (ESEM), while also allowing the estimation of several higher-order factors (i.e., work values factors) based on these first-order exploratory factors (Morin et al., 2016; see Figure S1 of the Online Supplements). ESEM appears to be an optimal analytical strategy to assess a first-order structure of work values. Factor scores can be extracted from an ESEM solution and used for subsequent analyses, such as EwC. Moreover, ESEM does not have to respect the over-restrictive requirement of assigning each item to a single factor (and constraining cross-loading to zero) when assessing multidimensional constructs like the first-order structure of work values, which could lead to inflated factor correlations and penalized goodness-of-fit indexes (Asparouhov & Muthén, 2009; Marsh et al., 2014). Three steps are needed to estimate an ESEM-within-CFA model: (1) The first-order structure (i.e., work values to items) is tested using an ESEM framework. Items freely loaded on their respective work value, whereas they were targeted to be as close as possible to zero to other factors. (2) The first-order ESEM structure obtained at step one is re-expressed in a confirmatory factor analysis (CFA) structure where all items load on all factors, by using the starting values generated from the final ESEM model. To obtain an over-identified model, items with the strongest factor loadings on their respective factor must be fixed on every first-order factor of the model (see Morin & Asparouhov, 2018). (3) The second-order structure (work values factors to work values) is expressed using a CFA.

Estimations of PNS and PNF at work were based on a bifactor ESEM model (B-ESEM; Morin et al., 2016) that included two global factors (g-factors; i.e., PNS and PNF at work) and three specific factors (s-factors; i.e., autonomy, competence, relatedness). Bifactor models are hierarchical structures allowing to separate the variance of multidimensional constructs between g- and s-factors (see Figure S2 of the Online Supplements). More specifically, g-factors explain the variance shared among all items of multidimensional constructs, whereas s-factors explain the covariance associated with specific factors that are not already explained by g-factors (Morin, 2016; Tóth-Király et al., 2018). Items freely loaded on their respective g-factor and s-factor, whereas they were targeted to be as close as possible to zero to other factors. Previous studies found B-ESEM to more adequately assess basic psychological needs (Tóth-Király et al., 2018), since it allows distinguishing variance from g- and s-factors. PNS and PNF at work factor scores were generated from the g-factors.

#### Latent profile analyses

Using factor scores obtained from measurement models, latent profile analyses (LPA) were carried to identify the best profile solution. Models including from one to eight profiles were estimated. To avoid convergence problems, all models were estimated using 5,000 random sets of starting values, 100 iterations to estimate the LPA models, and the 200 best solutions were retained for final stage optimization (Hipp & Bauer, 2006). Means and variances were also freely estimated in all profiles (Diallo et al., 2016). The choice of the optimal number of profiles was based on meaning and theoretical implications of the profiles (Morin, 2016), statistical adequacy of the solution (Bauer & Curran, 2004), and fit indices (Nylund et al., 2007).

Fit indices used to assess the quality of profile solutions were the Akaike Information Criterion (AIC), the Bayesian information criterion (BIC), the sample-adjusted BIC (ABIC), the adjusted Lo, Mendell, and Rubin's likelihood ratio test (aLMR), and the Bootstrap Likelihood Ratio Test (BLRT). A lower value on the AIC, BIC and ABIC suggests a better-fitting solution. However, these indicators can keep improving when adding another profile, without reaching a minimum value (Litalien et al., 2019). Therefore, AIC, BIC, and ABIC values were graphically presented in elbow plots. The optimal number of profiles is that after which the slope in the plot flattens (Morin et al., 2011). The aLMR and BLRT are used to compare model fit improvement by comparing solutions with *k* profiles and (*k* - 1) profiles. If the *p* value is not statistically significant, it indicates that the addition of a profile does not improve the model fit. In such cases, the *k* profile solution should be rejected in favor of a (*k* - 1) profile solution. The entropy value (varying from 0 to 1) was also examined for each solution, which represented how precise the classification of cases into profile is, with larger values indicating fewer classification errors (Morin, 2016). The entropy value should not be used to determine the optimal number of profiles (Lubke & Muthén, 2007).

#### Mean-level differences across profiles

Based on the solution retained, profiles were contrasted according to their mean levels of PNS and PNF at work. The BCH procedure in Mplus was used to compare means of continuous variables (i.e., PNS, PNF, age) across profiles by using a Wald χ^2^ test. The BCH procedure was preferred to other methods (e.g., DCON or manual three-step) because it avoids shifts in latent profiles when comparing mean levels on outcomes (Asparouhov & Muthén, 2020). Because *p* values have no interpretive value (Cumming & Calin-Jageman, 2017; Kline, 2013), the Wald χ^2^ values were used to calculate the effect size from group comparisons with Cohen’s *d*. Only effect size coefficients were considered for interpretation purpose, by using Cohen (1988) thresholds in which the strength of mean differences can be qualified as small (.2), moderate (.5), or strong (.8). The DCAT procedure in Mplus was used to compare the distribution of gender participants across profiles. Effect sizes were calculated with Cramer's *v*, where the strength of differences can be qualified as small (.1), moderate (.3), or strong (.5).

## Results

### Preliminary measurement models

Three models were tested to create factor scores of variables for subsequent analyses. First, the EwC model for work values revealed excellent fit to the data (χ^2^ = 2649.55, df = 1553, *p* < .001; CFI = .93; TLI = .90; RMSEA = .04; SRMR = .04). Factor loadings for the second-order model of work values are presented in Table S2 of the online supplements. Second, B-ESEM model for basic psychological needs at work yielded acceptable fit to the data (χ^2^ = 298.30, df = 163, *p* < .001; CFI = .94; TLI = .90; RMSEA = .04; SRMR = .03), after removing an item for autonomy satisfaction that did not load on its expected factor. Factor loadings for the B-ESEM model are presented in Table S3 of the online supplements. Overall, obtained solutions for every measurement model showed excellent to acceptable fit to the data, supporting the measurement adequacy of the scales used to assess the study’s constructs. Means, standard deviations, and correlations among factor scores are presented in Table 1.

### Profiles of work values

**Figure 2.**
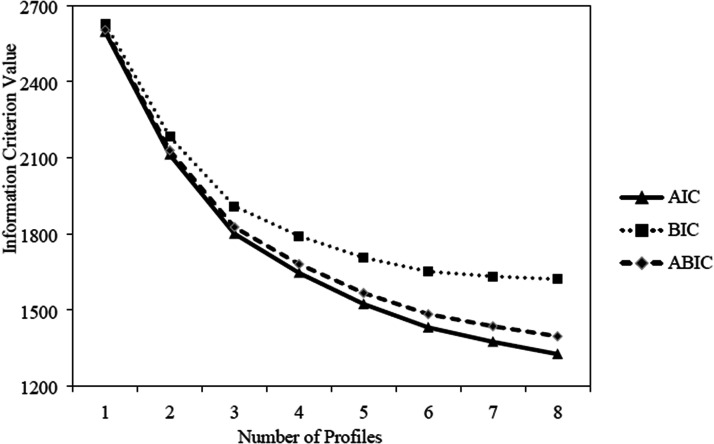
Elbow plot for the information criterion. *Note*. AIC: Akaike Information Criterion; BIC: Bayesian Information Criterion; ABIC: sample-adjusted BIC.

**Figure 3.**
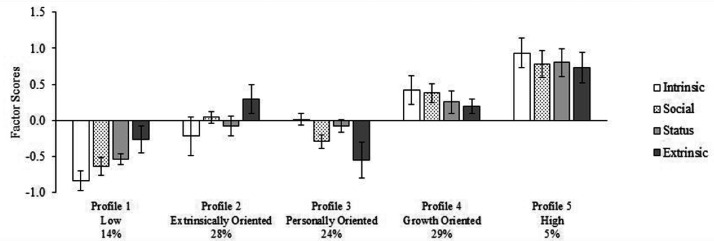
Final latent profile solution (N = 476).

LPA with one to eight profiles were estimated, for which fit indices are presented in Table 2. The information criteria (i.e., AIC, BIC, and ABIC) values did not reach a minimum after the addition of another solution, as observed by the elbow plots displayed in Figure 2. The elbow plot of information criteria suggests that a plateau is reached at five profiles, whereas the aLRT indicates that a four-profile solution is the best fitting solution. The BLRT was statistically significant for each solution. The four-profile solution was not well defined qualitatively and was only varying quantitatively (i.e., profiles with similar levels of valuing for all work values). The five-profile solution revealed that profiles were all well defined quantitatively and qualitatively and that they were distinct and theoretically meaningful. Therefore, the five-profile solution was retained (see Figure 3). Values of within-profile means and variances for each profile are reported in Table S4 of the online supplements. This solution showed a good level of classification accuracy, with an entropy value of 0.839.

**Table 2.** Results from latent profiles analyses (N = 476).

Model	LL	#fp	Scaling	AIC	BIC	ABIC	Entropy	aLMR	BLRT
1 profile	−1290.388	8	1.15	2596.777	2630.100	2604.709	.987	Na	Na
2 profiles	−1039.143	17	1.33	2112.285	2183.097	2129.142	.799	.002	≤.001
3 profiles	−874.422	26	1.24	1800.844	1909.145	1826.624	.801	.003	≤.001
4 profiles	−788.070	35	1.16	1646.141	1791.93	1680.845	.838	.002	≤.001
5 profiles	−717.656	44	1.26	1523.312	1706.591	1566.941	.839	.273	≤.001
6 profiles	−662.476	53	1.17	1430.951	1651.718	1483.504	.846	.105	≤.001
7 profiles	−625.159	62	1.38	1374.318	1632.574	1435.794	.870	.649	≤.001
8 profiles	−591.916	71	1.13	1325.831	1621.576	1396.232	.890	.074	≤.001

*Note*. LL: Model LogLikelihood; #fp: Number of free parameters; Scaling: Scaling factor associated with MLR loglikelihood estimates; AIC: Akaïke Information Criteria; BIC: Bayesian Information Criteria; ABIC: Sample-Size Adjusted BIC; aLMR: adjusted Lo, Mendell, and Rubin's Likelihood Ratio Test; BLRT: Bootstrap Likelihood Ratio Test.

Profile 1 (14% of the sample) characterizes participants reporting low levels of endorsement of intrinsic, social, and status work values and moderately low endorsement of extrinsic work values. This profile was labelled “Low” because it groups participants with the lowest levels of endorsement for most work values in the sample. Profile 2 (28% of the sample) included participants reporting moderately high levels of extrinsic work values, moderate levels of social and status work values, and moderately low levels of intrinsic work values. This profile was labelled “Extrinsically Oriented”, considering that these participants endorsed extrinsic work values more strongly than other work values. Profile 3 (24% of the sample; “Personally Oriented”) included participants who endorsed personal work values more strongly than external values. Profile 4 (29% of the sample) was labelled “Growth Oriented” and groups participants that endorsed growth work values (i.e., intrinsic and social) more strongly than instrumental work values (i.e., extrinsic and status). Profile 5 (5% of the sample) was labelled “High” and included participants who strongly endorsed intrinsic, social, and status work values but moderately endorsed extrinsic work values.

### Contrasting profiles

First, profiles were contrasted on two sociodemographic covariates, namely age and gender (see Tables 3 and 4). When examining participants’ age as a function of profile membership, pairwise comparisons revealed that participants from the High profile were younger on average than participants from the Low profile. The effect size for this difference was small. Other pairwise comparisons showed no substantial differences among profiles regarding participants’ average age based on effect size coefficients. Furthermore, pairwise comparisons regarding the gender distribution showed that the Low profile included the lowest proportion of women and the Extrinsically Oriented profile was the one with the highest proportion of women, when compared to other profiles. The effect sizes for these differences were small. The remaining profiles (Personally Oriented, Growth Oriented, and High) included a similar proportion of men and women.

**Table 3.** Standardized profile means and standard error of covariates (N = 476).

	Standardized profile means (standard error)				
	Profile 1 *Low* (14%)	Profile 2 *Extrinsically oriented* (28%)	Profile 3 *Personally oriented* (24%)	Profile 4 *Growth oriented* (29%)	Profile 5 *High* (5%)
Age	43.66 (1.28)	43.32 (1.11)	43.27 (1.01)	42.90 (0.90)	41.34 (2.31)
Gender (female)	57.4% (0.07)	79.7% (0.05)	68.6% (0.06)	70.2% (0.05)	68.2% (0.12)
PNS at Work	−0.24 (0.12)	−0.16 (0.11)	0.07 (0.10)	0.15 (0.09)	0.30 (0.09)
PNF at Work	−0.05 (0.13)	0.14 (0.11)	0.02 (0.11)	−0.10 (0.08)	−0.13 (0.20)

*Note*. PNS: Psychological Need Satisfaction; PNF: Psychological Need Frustration.

**Table 4.** Comparing profiles on needs and demographic variables.

	Effect sizes values for profile comparisons									
	1 vs 2	1 vs 3	1 vs 4	1 vs 5	2 vs 3	2 vs 4	2 vs 5	3 vs 4	3 vs 5	4 vs 5
Age	.03	.04	.07	.21	.00	.04	.16	.03	.17	.14
Gender (female)	.24	.12	.13	.10	.13	.11	.10	.02	.00	.02
PNS at work	.07	.31	.39^*^	.76^*^	.21	.28^*^	.57^*^	.07	.31	.21
PNF at work	.17	.06	.05	.08	.10	.22	.25	.11	.13	.03

*Note*. Size of differences between profiles are measured by Cohen’s *d* (PNS and PNF at work, and age) and Cramer’s *v* (gender) effect size. Cohen’s *d* effect size = .2 (small), .5 (moderate), .8 (strong). Cramer's *v* effect size = small (.1), moderate (.3), and large (.5). PNS = Psychological Need Satisfaction; PNF = Psychological Need Frustration.

^*^p < .05.

Profiles were then compared on measures of PNS and PNF at work. Profile means, standard errors, and effect size values are presented in Tables 3 and 4 and in Figure S3 of the Online Supplements. For PNS at work, pairwise comparisons showed that participants belonging to the High profile reported their psychological needs as being more strongly met at work than participants from the other profiles. The effect size of these differences was small for comparisons with Growth and Personally Oriented, moderate with Low, and moderately high with Extrinsically Oriented profiles. Also, participants belonging to Growth and Personally Oriented profile reported higher levels of PNS at work compared with participants from Extrinsically Oriented and Low profiles. The effect sizes for these differences were small. Finally, levels of PNS were similar for participants in Extrinsically Oriented and Low profiles as well as for those from the Growth and Personal Oriented profiles. Overall, these results showed that the level of PNS at work is mostly associated with the endorsement level of intrinsic work values. However, the endorsement level of social and status work values also appears to be partially related to need satisfaction at work. For PNF at work, mean differences indicated that participants in the Extrinsically Oriented profile reported being more frustrated than participants from the High and Growth Oriented profiles, although the effect size was small. Other pairwise comparisons showed no substantial differences among mean level of PNF based on effect size coefficients.

## Discussion

The goal of this study was to identify profiles of work values in adult workers and their association with PNS and PNF at work. Five profiles were identified (Low, Extrinsically Oriented, Personally Oriented, Growth Oriented, and High), in line with our hypothesis that there would be few (4 or 5) distinct profiles. The second goal of this study was to associate membership to work values profiles with measures of PNS and PNF at work. It was expected that participants belonging to profiles characterized by high levels of intrinsic and social work values and low levels of extrinsic and status would report higher levels of PNS at work and lower PNF at work. Results partially supported our hypothesis, as valuing intrinsic, social, and status work values was more related to a higher level of PNS at work, regardless of extrinsic work valuing. Regarding PNF at work, it was expected that participants in profiles characterized by low levels of intrinsic and social work values and high levels of extrinsic and status would report higher levels of PNF at work. Results also partially supported our hypothesis where participants reporting higher level of need frustration at work were those who reported endorsing extrinsic work values more strongly than they endorsed intrinsic, social, and status work values. These results have important scientific and applied implications, which are discussed in the next sections.

### Implications for theories and research

These results align with those from previous studies (Guo et al., 2018; Koh, 2016) by identifying distinct work values profiles using latent profile analysis. However, this study is the first to identify distinct and meaningful profiles of work values that are based on the FFM-WV. These profiles varied both quantitatively (general level of valuing of all work values) and qualitatively (distinct patterns of valuing work values factors). Therefore, this study showed that distinct groups of adult workers have unique combinations of work values. Furthermore, the five-profile solution was generally independent from sample characteristics, although few differences were observed. For instance, age was not a function of profile membership, although a small difference was observed where participants from the High profile were younger than those from the Low profile. Regarding gender, participants from three profiles (i.e., High, Personal, and Growth Oriented) reported similar distributions of women and men. The Low profile included the smallest number of women, whereas the Extrinsically Oriented profile included the highest proportion of women. However, multiple-group analysis of similarity would be needed to support the independence of the profile solution based on sample characteristics like gender or age, and provide evidence about cross-sample generalizability of the obtained (Morin et al., 2016). These analyses require a larger sample than the one used in the present study, which precluded us to further investigate multiple group similarity of the profile solution.

This study also showed that intraindividual patterns of work values are useful to understand workers’ levels of need satisfaction and frustration at work, which are fundamental to their functioning and thriving at work. Hence, these results support the SDT postulate (Kasser, 2002; Ryan et al., 1996) that growth-oriented (i.e., intrinsic and social) work values are positively related to a higher level of PNS at work. However, the present study showed that workers’ PNS at work was mostly a function of their endorsement of growth-oriented work values. For instance, even though participants from the High profile (who reported the highest levels of PNS at work compared to participants from other profiles) were reporting the highest level of extrinsic work values, they also reported the strongest endorsement of growth-oriented values. In other words, even if workers were strongly valuing instrumental work values (e.g., income or status) that are not aligned with psychological thriving, their needs were still satisfied, which could be attributed to their strong endorsement of growth-oriented work values. One possible explanation is that growth-oriented work values have a protective effect over valuing extrinsic work values. When workers value intrinsic work values to a lesser extent, they seem to endorse extrinsic work values more, which was the case for participants’ belonging to Low and Extrinsically Oriented profiles. In these situations, the possibly protective effect of intrinsic work values seems to vanish and lower level of PNS at work are observed. These findings are in line with previous findings showing the benefits to valuing intrinsic over extrinsic work values, such as being more effective (Deci et al., 2017 ), more flexible at work (Van den Broeck et al., 2010), and less emotionally exhausted in learning situations (Van den Broeck et al., 2011).

Unexpectedly, results exposed that workers’ PNS at work was also a function of status work values, but in a lesser extent than growth-oriented work values. In the FFM-WV, status work values are expected to be instrumental and to negatively relate to PNS at work, rather than being positively related to PNS, as observed in the correlation matrix. A possible explanation might be that some work values (i.e., recognition and advancement) included in this factor could contribute positively to PNS. By endorsing work values that promote the importance of being recognized for the work tasks they accomplished and the importance of having opportunities for career advancement, workers might fulfill their need of competence by feeling capable and effective in their actions. Therefore, examining the unique contribution of each psychological need could improve our understanding of their association with work values. However, status work values in the FFM-WV was previously shown to be unrelated to PNS at work and to contribute positively to PNF at work (Busque-Carrier et al., 2021b). Future research is needed to examine the association of status work values with PNS and PNF at work.

With respect to how profile membership was differentially associated with PNF at work, results showed an opposite pattern to that for PNS. That is, profiles more oriented toward extrinsic versus other work values included participants reporting the highest levels of frustration at work. However, this association is true under two conditions. First, participants from the Extrinsically Oriented profile reported the highest levels of need frustration at work. One possible explanation of this result might be that these participants were valuing extrinsic work values more than they valued other work values factors. Second, it might be argued that participants from the Low profile should have been the most frustrated participants. However, work values scores are described with factor scores, which represent endorsement level of each work values compared to other participants from this study. Therefore, participants from the Low profile are characterized by individuals that expressed lower endorsement for every work value, including extrinsic.

Overall, these results showed that participants from profiles reporting a high level of intrinsic and, in a lesser extent, social and status work values were those whose needs were most satisfied at work, whereas participants from profiles reporting lower levels of intrinsic work values and higher levels of extrinsic work values than the average were the most satisfied. Although additional research is needed, organizations and professionals could use these five profiles to identify which work values profile a worker falls in, which helps understand which criteria or orientations they endorse and how this configuration of work values dimensions promotes or thwarts their psychological needs at work. Strategies could thus be implemented to enhance valuing of intrinsic and social work values and to decrease endorsement of extrinsic work values for employees who belong to suboptimal profiles (i.e., Low and Extrinsically Oriented). For example, there is research showing that work environments can help shape work values (Zhang et al., 2019). More specifically, of the extent to which one endorses intrinsic values can be partially predicted by how supervisors adopt autonomy-supportive behaviors (i.e., providing meaningful rationale for requested tasks, emphasizing choice rather than control, and acknowledging employees’ feelings and perspectives; Hardré & Reeve, 2009). In contrast, workers’ endorsement of extrinsic values can be partially predicted by how their supervisors adopt controlling behaviors (i.e., neglecting or frustrating employees’ motivation, pressuring employees to behave in a specific way; Hardré & Reeve, 2009). Therefore, when workers prioritize extrinsic over intrinsic and social work values, trying to provide them with a more autonomy-supportive environment could contribute to increase their endorsement of intrinsic and social work values while decreasing extrinsic ones.

### Strengths, limits, and future research

In addition to the strengths of the study (i.e., using a validated work values model, estimating profile with LPA, use of factor scores which partially controlled measurement error), some limitations need to be considered when interpreting the findings. A first limit pertains to the correlations among the four work values. The correlation matrix showed that intrinsic work values were highly correlated to social and status work values. As previously mentioned, work values scores are expected to be related, because individuals who find work important will generally value every work values highly (Schreurs et al., 2014). However, the strength of their associations is expected to be moderate (Wöhrmann et al., 2016) rather than strong, as observed in this study. These correlations might explain why these two work values factors were similarly endorsed within most profiles. One possible explanation regarding these correlations might be explained by the analyses chosen to create work values scores. Previous studies showed that moderate to high correlations are expected in a hierarchical CFA, because when cross-loadings are set to zero, it inflates factor correlations (Asparouhov & Muthén, 2009; Marsh et al., 2014). To our knowledge, this study is the first to estimate work values with a hierarchical measurement model. Similar results have been obtained with hierarchical structure of general values, where some correlations among value factors were above .65 (Cieciuch et al., 2014). A second limit involves the sample characteristics, which limits the generalizability of these results. Participants came from a privileged background, as their income and education level were substantially higher than that of the average Canadian population. Previous studies on general values showed that individuals coming from less privileged background have more restricted access to individual resources, which can thwart the development of emancipation or growth values (Fischer et al., 2011; Welzel et al., 2003). Considering that socioeconomic status might have a similar influence on work values, future replication studies should specifically aim to include workers from more diverse backgrounds. Participants were also mostly experienced workers. Several studies showed generational differences of mean level of work values factors (Cogin, 2012; Twenge et al., 2010). Investigating these differences would be relevant for attracting and retaining at work the younger generation of workers. Therefore, future research should also aim to include younger workers. A last limit concerns the descriptive nature of the present study, which precludes drawing causal conclusions about the relation between variables of the study.

One suggestion for future research is to replicate the profiles obtained in the present study with a non-French Canadian sample. By replicating these results in other languages and cultures, it would lead to conclude that these profiles are not culture-specific. Until their replication, the latent profile solution has to be interpreted as preliminary (Morin et al., 2011). Another suggestion would be to replicate obtained profiles using a longitudinal design, by examining the stability of these profiles over time (i.e., latent transition analysis) or developmental patterns relating to the work values system (i.e., growth mixture modeling analyses). Finally, future studies should validate and further examine how work values profiles are associated to other important variables related to work settings behaviors, like work motivation and work engagement. These investigations could further support the interpretation that some profiles (e.g., High and Growth Oriented) are more optimal for psychological growth than others (e.g., Low and Extrinsically Oriented).

## Supplemental Material

sj-pdf-1-prx-10.1177_00332941211040439 - Supplemental material for Linking Work Values Profiles to Basic Psychological Need Satisfaction and Frustration Supplemental material, sj-pdf-1-prx-10.1177_00332941211040439 for Linking Work Values Profiles to Basic Psychological Need Satisfaction and Frustration by Mathieu Busque-Carrier, Catherine F. Ratelle and Yann Le Corff in Psychological Reports
