# Supplementary material for: Linking Work Values Profiles to Basic Psychological Need Satisfaction and Frustration
Source: Psychol Rep. 2021 Aug 14;125(6):3183–208. doi: 10.1177/00332941211040439 (PMC9578103; doi:10.1177/00332941211040439)

Online Supplementary Materials for:

Linking Work Values Profiles to Basic Psychological Need Satisfaction and Frustration

Authors' Note:

These online appendices are to be posted on the journal website and hot-linked to the manuscript. If the journal does not offer this possibility, these materials can alternatively be posted on one of our personal websites (we will adjust the in-text reference upon acceptance).
These materials are provided for readers who want more information about the measurement models used or the within-profile means and variance of the final profile solution.

Sections

1. Table S1. Definitions of Work Values and Sample Items.
2. Table S2. Factor Loadings from the Four-Factor ESEM-Within-CFA Model of Work Values (N = 476).
3. Table S3. Factor Loadings for the Bifactor-ESEM Model of Basic Psychological Needs at Work (N = 433).
4. Table S4. Within-Profile Means and Variance for the Five-Profile Solution (N = 476).
5. Figure S1. Simplified Representation of an ESEM-within-CFA Model.
6. Figure S2. Simplified Representation of a Bifactor ESEM Model of Need Satisfaction and Frustration at Work.
7. Figure S3. Factor Scores and 95% Confidence Intervals of Psychological Need Satisfaction and Frustration at Work for the Five-Profile Solution (N = 476)

Table S1

Definitions of Work Values and Sample Items

Work values	Definition [...] = Importance given to…	Sample item [...] = At work, it is important for me…
Achievement	[...] to feel a sense of accomplishment at work.	[...] to see the results of my efforts.
Advancement	[...] the possibility of advancement opportunities, reaching a higher position, and increasing their responsibilities at work.	[...] to have opportunities for career advancement.
Aesthetics	[...] the presence, use and creation of products, and services that are attractive and aesthetically appealing.	[...] to use my imagination to create new products.
Altruism	[...] help others at work or to promote their well-being.	[...] to be of service to others.
Authority	[...] being in a position of authority at work, whether in planning, organizing, or carrying out the work of others.	[...] to lead the work of others.
Autonomy	[...] choosing how tasks are performed, without being supervised.	[...] to choose my work methods.
Benefits	[...] a job offering benefits, such as vacation weeks, sick leave, insurance programs and a good pension plan.	[...] to have a job with paid sick days.
Challenge	[...] a job in which the tasks to be performed are challenging in terms of knowledge, skills, and abilities.	[...] to surpass myself.
Colleagues	[...] a job where relations with co-workers are friendly and harmonious.	[...] to maintain positive interactions with my colleagues.
Creativity	[...] the possibility of using one's creativity in preparing and carrying out tasks.	[...] to use my creativity.
Development of Abilities	[...] a job in which it is possible to develop skills and abilities, and learn in a continuous fashion.	[...] to be able to improve my abilities.
Income	[...] a job where the salary is high, allowing oneself to be financially wealthy.	[...] to have a good salary.
Independence	[...] being independent in performing one’s professional tasks, like having like its own business.	[...] to make my own decisions.

Influence	[...] the possibility of exercising influence over others and the organization in general.	[...] to influence my colleagues' view.
Intellectual Stimulation	[...] a work in which it is possible to solve new problems, where it is necessary to be alert mentally, and which requires an intellectual effort.	[...] that tasks' completion requires a high level of thinking.
Prestige	[...] a work which arouses the admiration of others by its importance, which is well seen by others.	[...] to be the leader of the team.
Recognition	[...] work in which it is possible to be recognized for a job well done or for services rendered.	[...] to be recognized for the work tasks that I accomplished.
Security	[...] a job in which it is possible to work for as long as desired.	[...] to have work security.
Supervisors	[...] a job where the boss treats his employees well and equally.	[...] to have an understanding boss.
Travel	[...] a work that allows foreign travel and seeing the world.	[...] to travel as part of my job.
Use of Abilities	[...] a job where one's can fully use its skills and abilities.	[...] to perform tasks for which I have been trained.
Variety	[...] given to a job where the work tasks are diversified.	[...] to perform diversified tasks.
Work Environment	[...] a job in which the working environment is sheltered from bad weather and comfortable.	[...] to work in a lit environment.
Work-Life Balance	[...] a job that allows a good balance between professional and personal life domains.	[...] to have enough time for my hobbies.

Note. Sample items were originally created in French and translated for illustrative purposes.

Table S2

Factor Loadings for the Four-Factor ESEM-Within-CFA Model of Work Values (N = 476).

	Factors			
Work Values	Intrinsic	Extrinsic	Social	Status
Variety	.68			
Intellectual Stimulation	.64			
Development	.64			
Creativity	.61			
Autonomy	.38			
Security		.71		
Income		.61		
Work Environment		.53		
Work-Life Balance		.42		
Supervisors			.61	
Altruism			.59	
Travel				.49
Recognition				.47
Advancement				.46
Authority				.45

Table S3

Factor Loadings for the Bifactor-ESEM Model of Basic Psychological Needs at Work (N = 433).

	G-Factors		S-Factors			Item Uniqueness
	Satisfaction	Frustration	Autonomy	Relatedness	Competence	
auto_satis1	**.42**		**.24**	-.12	-.27	.68
auto_satis2	**.59**		**.05**	-.19	-.16	.59
auto_satis3	**.62**		**-.01**	-.12	-.03	.60
auto_satis4	**.48**		**.12**	-.07	-.26	.68
auto_frust1		**.58**	**-.28**	.17	.17	.52
auto_frust2		**.44**	**-.56**	.02	.04	.50
auto_frust3		**.47**	**-.45**	.06	.23	.53
relat_satis1	**.47**		.03	**.38**	-.19	.60
relat_satis2	**.54**		.07	**.52**	-.02	.43
relat_satis3	**.55**		.10	**.59**	.08	.34
relat_satis4	**.42**		-.01	**.50**	-.07	.57
relat_frust1		**.53**	.14	**-.24**	.15	.62
relat_frust2		**.63**	.17	**-.20**	.17	.51
relat_frust3		**.68**	.27	**-.30**	.15	.36
relat_frust4		**.62**	.10	**-.46**	.11	.39
comp_satis1	**.58**		-.09	-.08	**.25**	.59
comp_satis2	**.49**		-.12	-.02	**.43**	.56
comp_satis3	**.60**		.09	-.04	**.35**	.51
comp_satis4	**.46**		-.02	-.05	**.37**	.65
comp_frust1		**.50**	.20	.21	**-.47**	.45
comp_frust2		**.54**	.22	.15	**-.26**	.57
comp_frust3		**.53**	.22	.17	**-.37**	.50
comp_frust4		**.48**	.31	.19	**.03**	.63

Note. Target loadings are shown in **bold**. Autonomy Frustration scale has only three items because one item did not load on its expected factor and was removed. Auto = Autonomy; Relat = Relatedness; Comp = Competence; Satis = Satisfaction; Frust = Frustration.

Table S4

Within-Profile Means and Variances for the Five-Profile Solution (N = 476).

	Factor Scores			
	Intrinsic	Extrinsic	Social	Status
Profile 1 – Low				
Mean	-0.84	-0.27	-0.64	-0.54
95% C.I.	[-0.70; -0.97]	[-0.08; -0.46]	[-0.51; -0.76]	[-0.47; -0.62]
Variance	0.15	0.42	0.19	0.05
Profile 2 – Extrinsic Oriented				
Mean	-0.22	0.30	0.04	-0.08
95% C.I.	[-0.49; 0.05]	[0.10; 0.50]	[-0.04; 0.12]	[-0.22; 0.05]
Variance	0.05	0.09	0.04	0.03
Profile 3 – Personal Oriented				
Mean	0.01	-0.55	-0.30	-0.08
95% C.I.	[-0.07; 0.10]	[-0.31; -0.80]	[-0.20; 0.39]	[-0.17; 0.01]
Variance	0.07	0.21	0.06	0.05
Profile 4 – Growth Oriented				
Mean	0.42	0.19	0.38	0.26
95% C.I.	[0.22; 0.62]	[0.09; 0.29]	[0.24; 0.51]	[0.09; 0.42]
Variance	0.05	0.17	0.04	0.04
Profile 5 – High				
Mean	0.93	0.73	0.78	0.80
95% C.I.	[0.73; 1.14]	[0.52; 0.94]	[0.60; 0.97]	[0.61; 0.99]
Variance	0.03	0.05	0.02	0.03

Note. CI = 95% Confidence Interval.

Figure S1

Simplified Representation of an ESEM-within-CFA Model

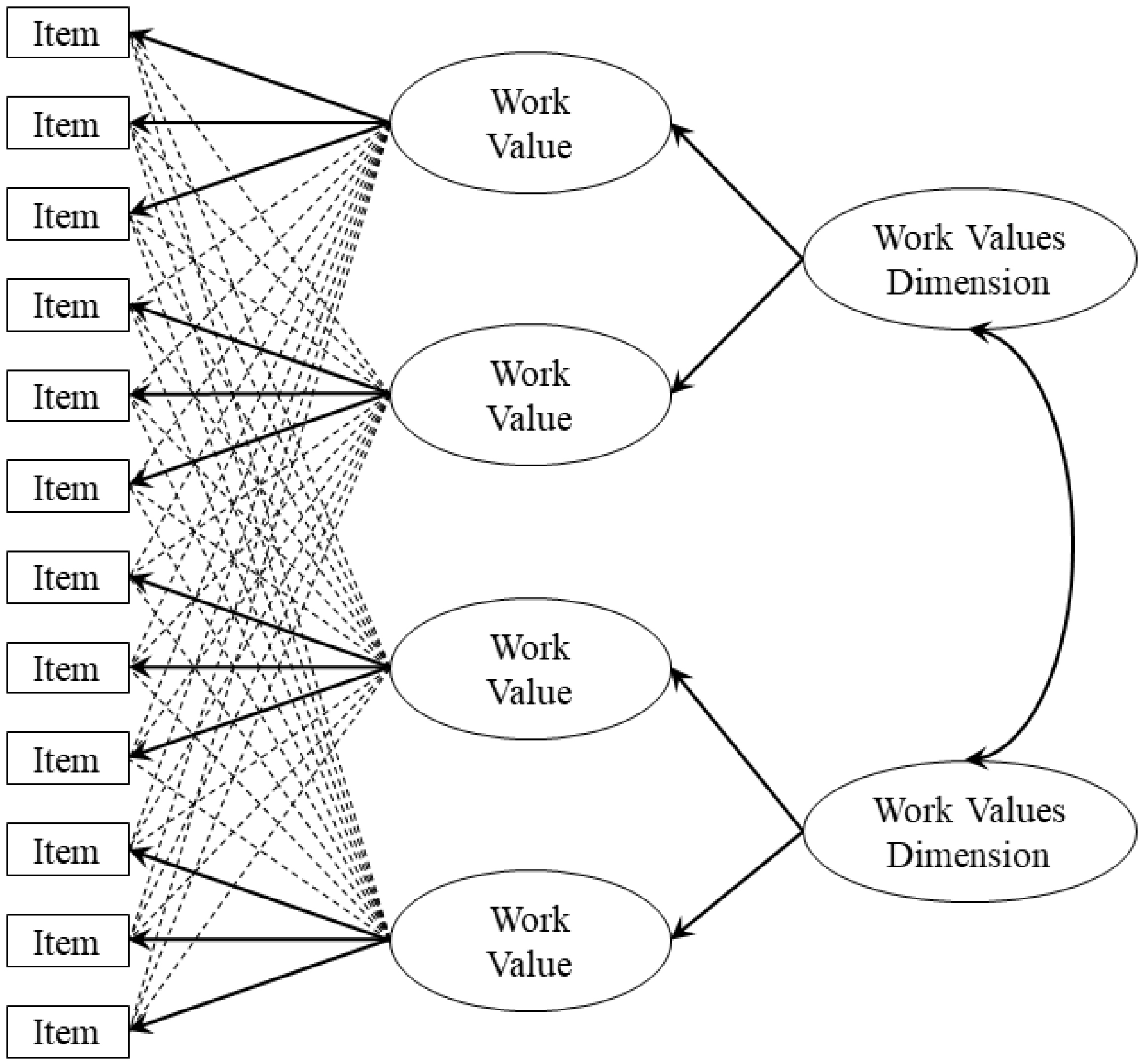

Note. Dotted lines represent item targeted to be as close as possible to zero.

Figure S2

Simplified Representation of a Bifactor ESEM Model of Need Satisfaction and Frustration at Work.

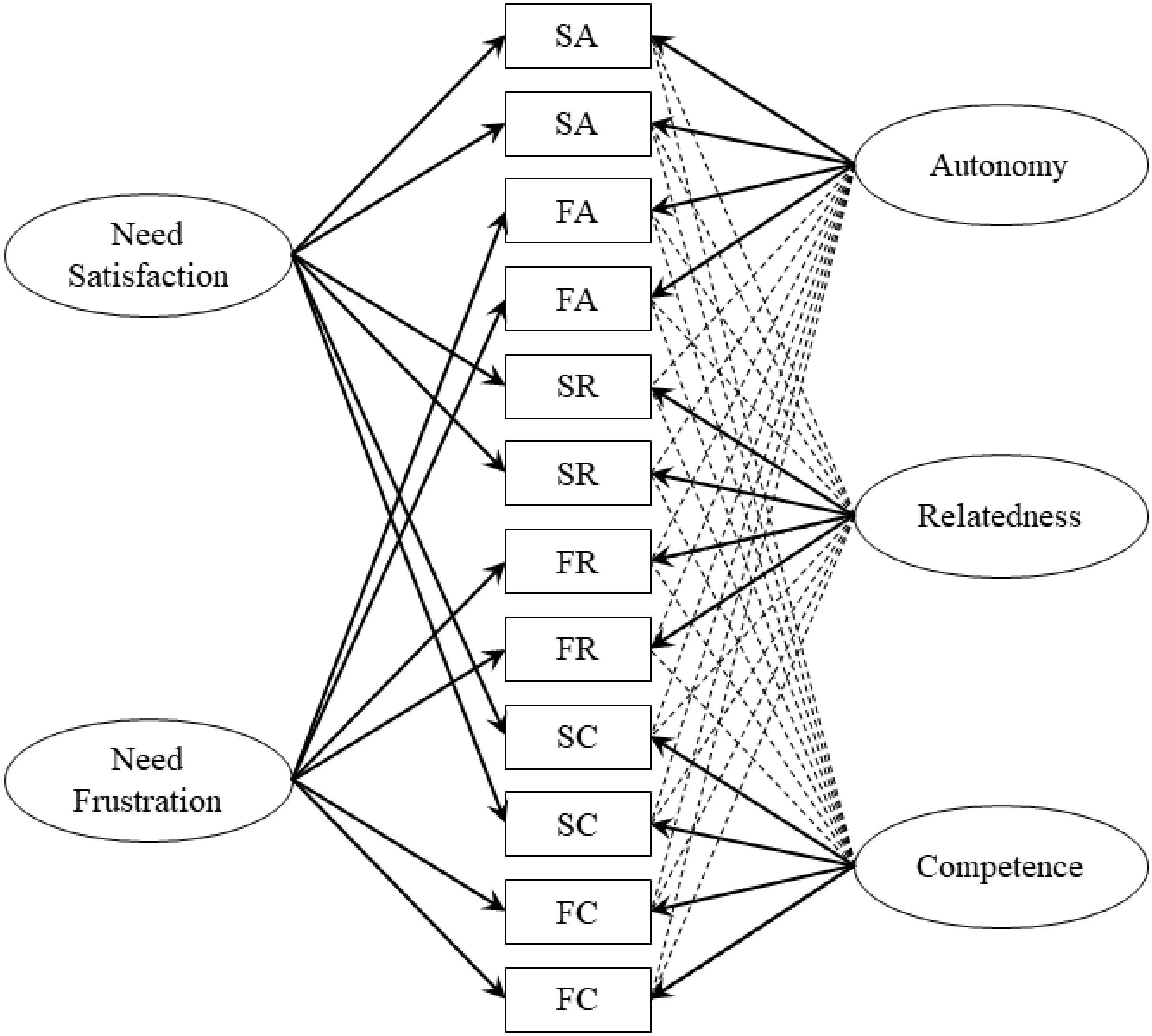

Note. Dotted lines represent item targeted to be as close as possible to zero. A = Autonomy; R = Relatedness; C = Competence; S = Satisfaction; F = Frustration.

Figure S3

Factor Scores and 95% Confidence Intervals of Psychological Need Satisfaction and Frustration at Work for the Five-Profile Solution (N = 476)

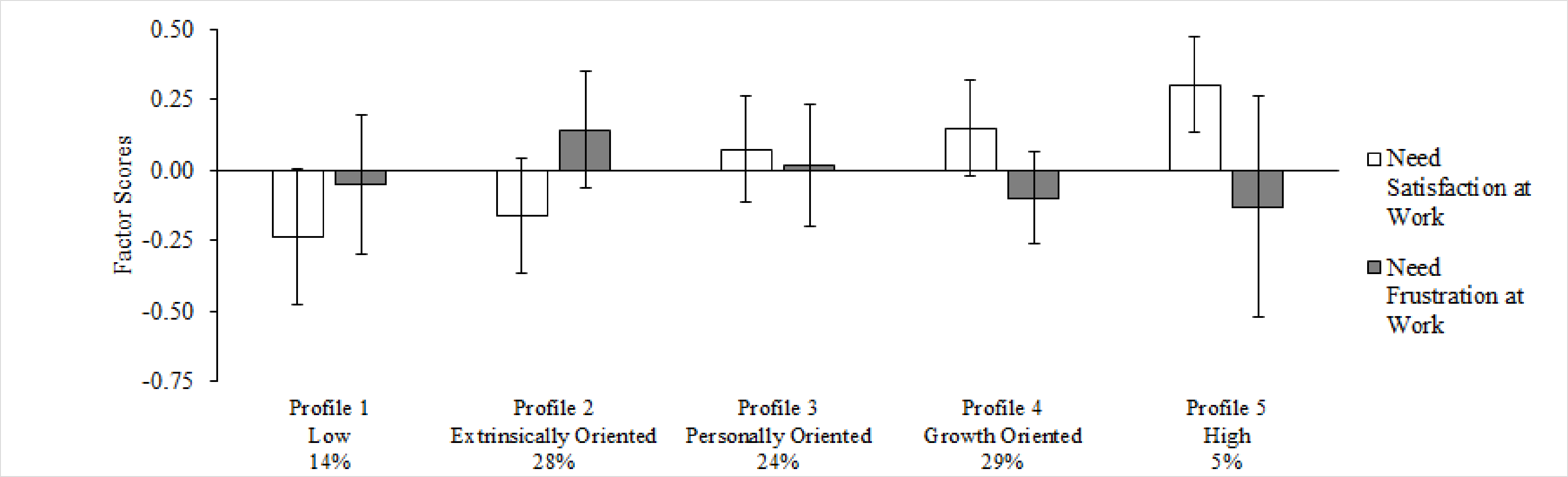

Supplement: sj-pdf-1-prx-10.1177_00332941211040439 - Supplemental material for Linking Work Values Profiles to Basic Psychological Need Satisfaction and Frustration [file sj-pdf-1-prx-10.1177_00332941211040439.pdf]
